# Observer agreement in single computerized tomography use for diagnosing paediatric head and neck malignancies at Uganda Cancer Institute

**DOI:** 10.1186/s43046-023-00179-y

**Published:** 2023-07-10

**Authors:** Alex Mwesigwa Mugisha, Zeridah Muyinda, Joyce Balagadde Kambugu, Denise Apolot, Elizabeth Atugonza, Anneth Teu, Aloysius Gonzaga Mubuuke

**Affiliations:** 1 Makerere University, College of Health Sciences, Kampala, Uganda; 2 Mulago National Referral Hospital, Kampala, Uganda; 3 Uganda Cancer Institute, Kampala, Uganda

**Keywords:** Paediatric head and neck malignancies, Radiation induced secondary malignancy, Computerized tomography, Observer agreement

## Abstract

**Background:**

In the Ugandan setting, investigation for PHNM with CT uses a protocol with both unenhanced and contrast enhanced procedures hence doubling the ionizing radiation exposure. The purpose of this study was to determine the feasibility of single CT procedures in diagnosing PHNM.

**Methods:**

This was a cross-sectional study using CT images from patients, aged fifteen years and below, investigated for head and neck malignancies at the Uganda Cancer Institute. Three radiologists, observers A, B and C, with 12, 5 and 2 years of experience, respectively, participated in the study. They independently reported contrast enhanced images (protocol A), unenhanced images (protocol B), then both unenhanced and contrast enhanced images (protocol C) in 2 months intervals. Inter- and intra- observer agreement was determined using Gwen’s Agreement coefficient.

**Results:**

Seventy-three CT scans of 36 boys and 37 girls, with a median age of 9 (3–13) years, were used. Intra-and inter-observer agreement on primary tumour location ranged from substantial to almost perfect with the highest intra-observer agreement observed when protocols A and C were compared. Inter-observer agreement for tumour calcifications was substantial for protocol A. Observers A and C demonstrated an almost perfect intra-observer agreement when protocols A and C were compared. There was a substantial inter-observer agreement on diagnosis for all protocols.

**Conclusions:**

In our setting and examining a limited number of CT images, we demonstrated that contrast-enhanced CT scans provide sufficient information with no evidence of additional value of unenhanced images. Using contrast-enhanced images alone reduced the radiation exposure significantly.

## Background

Paediatric head and neck malignancies (PHNM) are defined as cancers that occur, in the age range of 0 to 18 years, in the region between the skull base and the sternum which includes the orbits and paranasal sinuses [1].

The International Agency for Research on Cancer estimates that annually, about 9.4 incident malignancy cases per 100,000 occur in children below 15 years worldwide, with an age and sex adjusted mortality rate of about 5.4 cases/year per 100,000 [2]. A large proportion of this (85%) occurs in developing countries, and this was earlier estimated to increase within the next 20 years [3].

The use of computerized tomography (CT) during malignancy management is on the rise [4], and PHNM patients will at one time have radiological imaging to aid with diagnosis, influence treatment and monitor treatment response [5]. When using CT for PHNM diagnosis, either the unenhanced procedure, contrast enhanced procedure or a combination of both is performed and contrast enhanced CT procedures are recommended as they delineate the tumours, outline the borders, enable assessment of enlarged cervical lymph nodes and vascular abnormalities [6].

With the documented increase in incidence of PHNM, especially in Sub-Saharan Africa and an increase in use of CT for diagnosis, intervention, treatment response and surveillance, there is increased exposure to ionizing radiation and an increased lifetime potential risk for radiation-induced secondary malignancies (RISM), as low dose radiation has been associated with development of malignancy (RISM) [7, 8]. The lower the age of exposure to radiation, the higher the chances of RISM [9, 10]. Children have a life time risk of RISM that is up to ten times that of adults [11, 12], as malignancy radiation risks are present even after half a century of initial exposure [10].

Interventions, such as adjusting radiation dose due to size and age, have been put in place in order to reduce radiation dose to children when imaged with CT; however, the most effective way is to reduce the number of CT procedures one receives [13]. Elimination of double CT procedures as a means of dose reduction during paediatric imaging would require an observer agreement study to compare findings and agreement from unenhanced and contrast enhanced CT procedures with double CT procedures [14]. Imaging is increasingly used when gold standard validation is not available, such as in head and neck tumours where biopsies may not be obtained and in such circumstances, only observer agreement studies can assess the objectivity of imaging results [15].

There is a dearth of published literature especially from low resource settings documenting such observer agreements, which would inform the most effective management without unnecessary exposure to the potentially risky ionizing radiation from CT scans.

This observer agreement study highlights on the diagnostic information of PHNM and agreement values among different observers while using single CT procedures when compared to double CT procedures and proposes appropriate recommendations that can aid both radiologists and other clinicians when investigating these paediatric tumours. The purpose of this study therefore was to identify a single CT procedure sufficient in diagnosing paediatric head and neck malignancies, without loss of diagnostic information, in a bid to reduce the radiation burden and future risk of disease placed upon the patients.

## Methods

This was a cross-sectional descriptive study. The study was conducted over a period of 6 months at the Uganda Cancer Institute (UCI), the largest oncology treatment and research Centre in East Africa. Seventy-three archived head and neck CT images from the year 2016 to 2020 and belonging to subjects aged fifteen years and below were included in the study. CT images were determined using a stratified random sampling method. The inclusion criteria were as follows: subjects who were being investigated for head and neck malignancy with good diagnostic quality images and had both unenhanced and contrast enhanced CT procedures done. Exclusion criteria was subjects with prior intervention for the head and neck malignancy. A purposive typical case sampling method was used to determine three diagnostic radiologists as observers. Observers A, B and C had 12, 5 and 2 years of experience, respectively. The observers independently reported contrast enhanced images (protocol A), then unenhanced images (protocol B) and then both unenhanced and contrast enhanced images (protocol C) with 2 months intervals between reporting for each protocol. A head and neck structured template from Radiology Society of North America was used to document the image findings. Intra- and inter-observer agreements for primary tumour location, tumour calcifications, presence of lymphadenopathy and the first diagnosis were calculated using Gwet’s Agreement Coefficient. The strength of the agreement was defined as suggested by Landis and Koch: *κ* < 0, poor; 0–0.20, slight; 0.21–0.4, fair; 0.41–0.6, moderate; 0.61–0.8, substantial; and 0.81–1.0, almost perfect [16, 17].

The IBM SPSS Statistics 25 software was used for analysis of the study findings.

## Results

The aim of this study was to determine the intra- and inter-observer agreement between paediatric head and neck malignancy findings of single CT procedures and double CT procedures for children aged 15 years and below at Uganda Cancer Institute.

Seventy-three (73) patients aged 15 years and below were included in this study. There were 36 (49.3%) boys with median age of 9 (3–13) years.

Observer A had 12 years of experience; observer B had 5 years of experience while observer C had 2 years of experience.

Inter-observer agreement among the three observers using contrast only CT procedures (Table 1).

**Table 1:** Inter-observer agreement when using protocol A (contrast enhanced CT only studies), protocol B (unenhanced CT scans) protocol C (both the unenhanced and contrast enhanced CT scans)

Characteristic	Agreement using protocol A	Gwet’s AC1 (95% CI)	*P* value	Agreement using protocol B	Gwet’s AC1 (95% CI)	*P* value	Agreement using protocol C	Gwet’s AC1 (95% CI)	*P* value
Primary tumour location	74.0 (65.9–82.1)	0.71 (0.62–0.80) Substantial	< 0.001	79.0 (70.8–87.3)	0.74 (0.67–0.85) Substantial	< 0.001	85.8 (79.3–92.4)	0.84 (0.76–0.92) Almost perfect	< 0.001
Tumour calcifications	77.2 (69.7–84.6)	0.7 (0.59–0.81) Substantial	< 0.001	87.7 (81.4–93.9)	0.83 (0.75–0.91) Almost perfect	< 0.001	86.8 (80.3–93.2)	0.84 (0.75–0.92) Almost perfect	< 0.001
Lymphadenopathy	71.7 (64.0–79.4)	0.47 (0.31–0.62) Moderate	< 0.001	84.5 (77.9–91.1)	0.69 (0.56–0.82) Substantial	< 0.001	89.0 (83.2–94.9)	0.79 (0.68–0.90) Substantial	< 0.001
Diagnosis	67.6 (58.7–76.5)	0.61 (0.51–0.72) Substantial	< 0.001	73.5 (64.6–82.4)	0.68 (0.58–0.79) Substantial	< 0.001	73.5 (64.6–82.4)	0.68 (0.56–0.79) Substantial	< 0.001

From Table 1, one can see that tumour calcifications were demonstrable on contrast enhanced CT only procedures, with a substantial agreement. A similar agreement was obtained for final diagnosis when compared with both unenhanced and contrast enhanced CT procedures.

Unenhanced CT only procedures are sufficient in determining a diagnosis, evidenced by the substantial agreement among the three observers, and they also compare to procedures with double CT procedures in determining presence of lymphadenopathy and calcifications.

Intra-observer agreement when contrast enhanced CT only procedure findings were compared to findings of both unenhanced and contrast enhanced CT procedures (Table 2).

**Table 2:** Intra-observer agreement using protocol A (contrast enhanced) and protocol C (both)

Characteristic	Observer A agreement (95% CI)	Gwet’s AC1 (95% CI)	*P* value	Observer B agreement	Gwet’s AC1 (95% CI)	*P* value	Observer C agreement	Gwet’s AC1 (95% CI)	*P* value
Primary tumour location	72.6 (62.1–83.1)	0.71 (0.57–0.80) Substantial	< 0.001	73.9 (63.7–84.3)	0.80 (0.70–0.90) Substantial	< 0.001	89.0 (81.7–96.4)	0.87 (0.79–0.96) Almost perfect	< 0.001
Tumour Calcifications	68.5 (57.6–79.4)	0.58 (0.42–0.74) Moderate	< 0.001	78.1 (60.4–87.8)	0.73 (0.60–0.86) Substantial	< 0.001	90.4 (83.5–97.3)	0.88 (0.79–0.97) Almost perfect	< 0.001
Lymphadenopathy	90.4 (83.4–97.3)	0.82 (0.69–0.95) Almost perfect	< 0.001	68.5 (57.6–79.4)	0.37 (0.15–0.59) Fair	< 0.001	89.0 (81.7–96.4)	0.81 (0.68–0.94) Almost perfect	< 0.001
Diagnosis	73.2 (62.3–84.2)	0.68 (0.55–0.81) Substantial	< 0.001	64.4 (53.1–75.6)	0.57 (0.44–0.71) Moderate	< 0.001	91.8 (85.3–98.2)	0.90 (0.85–0.98) Almost perfect	< 0.001

One can generally conclude from Table 2 that there is better agreement among the individual observers when findings on contrast enhanced CT only procedures as opposed to those on unenhanced CT only procedures are compared with findings on procedures with both unenhanced and contrast enhanced CT procedures.

Intra-observer agreement when unenhanced CT only procedure findings were compared to findings of both unenhanced and contrast enhanced CT procedures (Table 3).

**Table 3:** Intra-observer agreement using protocol B (unenhanced) and protocol C (both)

Characteristic	Observer A agreement (95% CI)	Gwet’s AC1 (95% CI)	*P* value	Observer B agreement	Gwet’s AC1 (95% CI)	*P* value	Observer C agreement	Gwet’s AC1 (95% CI)	*P* value
Primary tumour location	76.7 (66.8–86.6)	0.73 (0.62–0.85) Substantial	< 0.001	71.2 (60.6–81.9)	0.67 (0.56–0.80) Substantial	< 0.001	73.6 (62.9–84.2)	0.71 (0.59–0.82) Substantial	< 0.001
Tumour calcifications	80.8 (71.6–90.1)	0.75 (0.63–0.88) Substantial	< 0.001	76.7 (66.8–86.6)	0.71 (0.57–0.84) Substantial	< 0.001	71.2 (60.6–81.9)	0.61 (0.46–0.76) Substantial	< 0.001
Lymphadenopathy	82.2 (73.3–91.2)	0.65 Substantial	< 0.001	75.3 (65.2–85.5)	0.51 Moderate	< 0.001	84.9 (76.5–93.3)	0.72 Substantial	< 0.001
Diagnosis	66.7 (55.4–77.9)	0.60 (0.47–0.74) Moderate	< 0.001	63.4 (51.6–75.1)	0.58 (0.44–0.71) Moderate	< 0.001	100	1 Almost perfect	< 0.001

From Table 3, it can be inferred that findings for primary tumour location and presence of tumour calcifications on unenhanced CT only procedures are comparable to findings on procedures with both unenhanced and contrast enhanced CT for each of the observers.

The imaging features of paediatric head and neck malignancies on unenhanced and contrast 146 enhanced head and neck CTs are shown in Figs. 1, 2, 3, 4, 5, 6, 7, and 8.

**Fig. 1:**
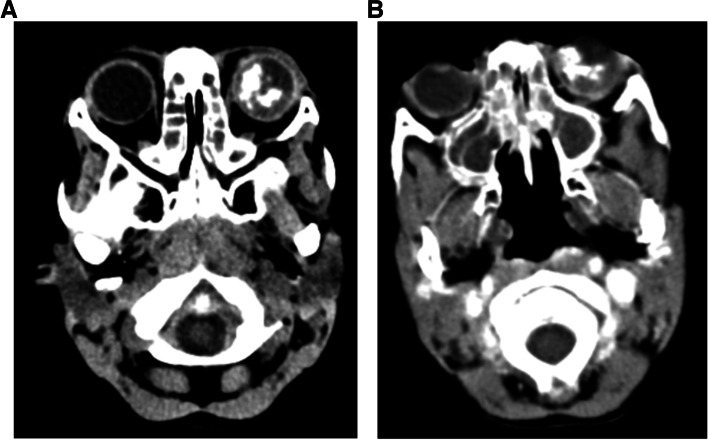
**A** An unenhanced axial CT image at the level of the orbits. There was a left orbital mass with calcifications involving the vitreous. The mass measured 2.1 × 1.8 cm. **B** A contrast enhanced axial CT, which demonstrated the same features as on **A**. There was no enhancement of the left vitreous mass. A diagnosis of a left orbital retinoblastoma was made on **A** and **B**

**Fig. 2:**
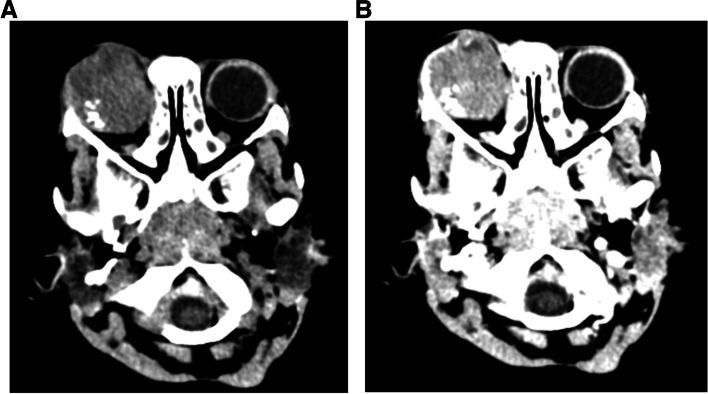
**A** An unenhanced axial CT at the level of the orbits. There was a large right intra-ocular mass, with calcifications, involving the anterior and posterior chambers. The mass measures 3.7 × 3.4 cm. **B** A contrast enhanced CT and demonstrated the same features as on **A**. There was no significant enhancement of the right ocular mass and the calcifications were visible. A diagnosis of right orbital retinoblastoma was made on **A** and **B**

**Fig. 3:**
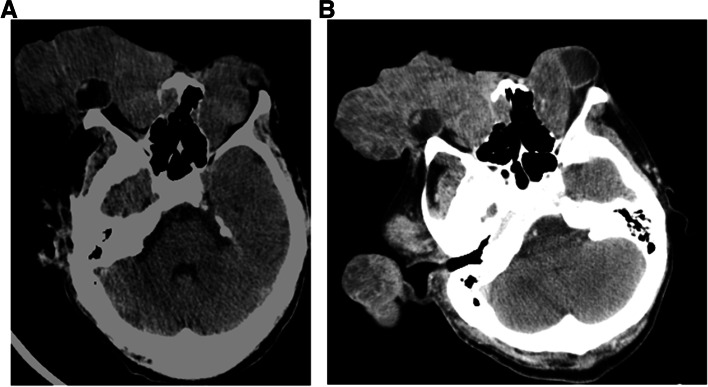
**A** An unenhanced axial CT at the level of the orbits. There was a large right extra-ocular mass involving the globe and ocular muscles. The mass measured 9.4 × 6.0 cm. There was a left orbital mass involving the left medial rectus muscle and it measured 5.4 × 2.8 cm. There was bilateral proptosis. There were no calcifications seen with in the masses. **B** A contrast enhanced axial CT and demonstrated all the features in **A** and also heterogeneous enhancement of the masses. A diagnosis of metastatic rhabdomyosarcoma was made on both **A** and **B**

**Fig. 4:**
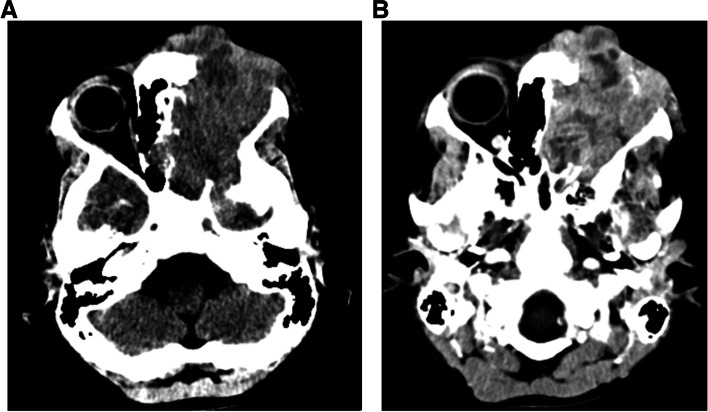
**A** An unenhanced axial CT at the level of the orbits. There was a large iso-dense mass with in the left orbit, involving the ocular muscles and globe. The mass measured 5.0 × 6.0 cm and had necrotic areas. There was extension to the middle cranial fossa. There were no calcifications seen within the mass. **B** A contrast enhanced axial CT and demonstrated heterogeneous enhancement of the left orbital mass. A diagnosis of left orbital rhabdomyosarcoma was made on both **A** and **B**

**Fig. 5:**
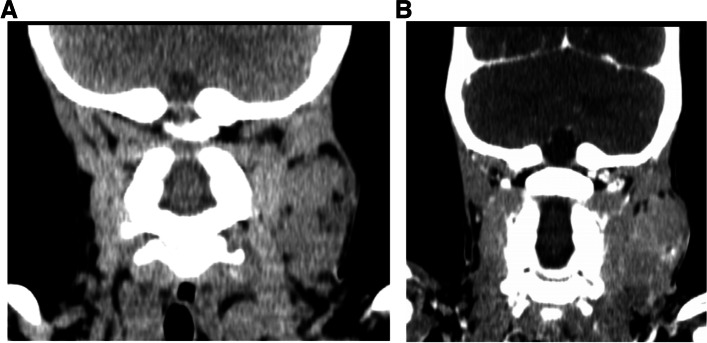
**A** An unenhanced coronal CT of the head and neck. There was multiple enlarged left level II, III, IV and V lymph nodes of up to 3.5 cm. **B** A contrast enhanced coronal CT, which demonstrated enlarged poorly enhancing left level II, III, IV and V lymph nodes. A diagnosis of lymphoma was made on both **A** and **B**

**Fig. 6:**
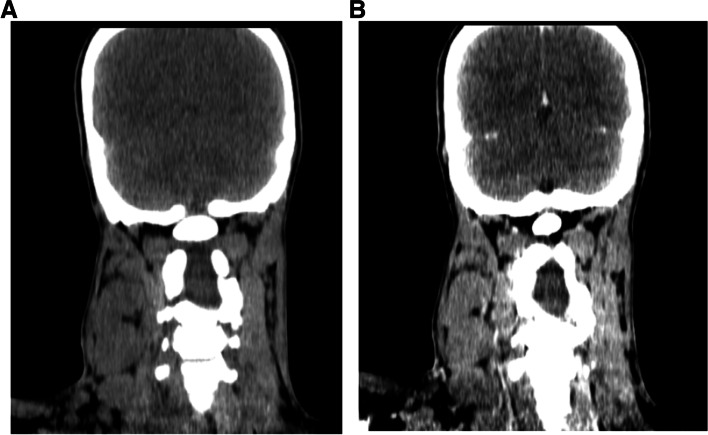
**A** An unenhanced coronal CT of the head and neck. There were multiple enlarged right level II, III, IV and V lymph nodes measuring up to 6 cm. **B** A contrast enhanced coronal CT, which demonstrated poorly enhancing right level II, III, IV and V lymph nodes. A diagnosis of lymphoma was made on both **A** and **B**

**Fig. 7:**
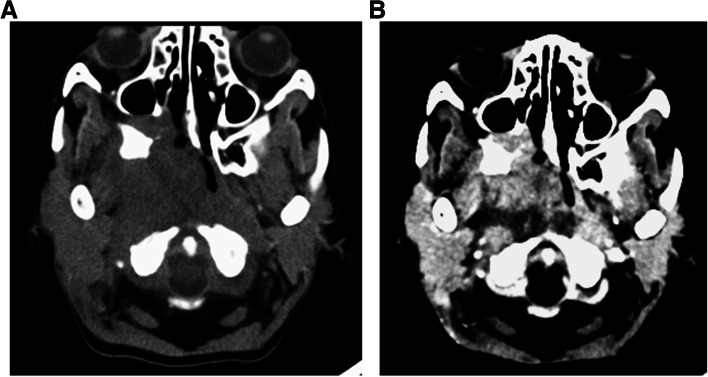
**A** An unenhanced axial CT at the level of the orbits. There was an iso-dense mass with in the nasopharyngeal space. The mass measured 4.5 cm × 3.4 cm. There were no calcifications seen with in the mass. **B** A contrast enhanced axial CT at the same level. The mass demonstrated heterogeneous enhancement. This was a diagnosis of nasopharyngeal carcinoma, made on both **A** and **B**

**Fig. 8:**
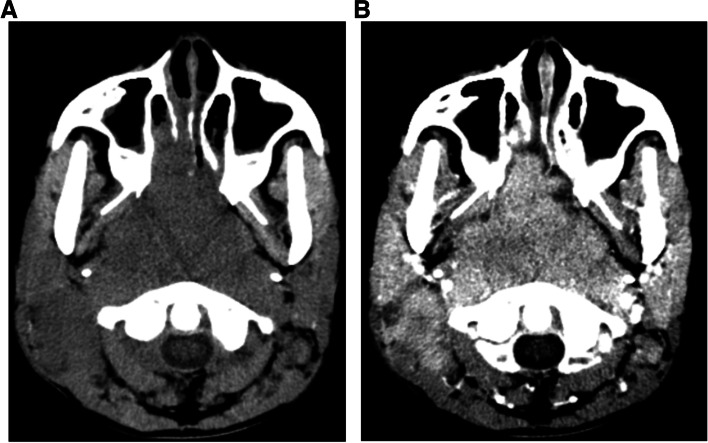
**A** An unenhanced axial CT at the level of the maxillary sinuses. There was a large iso-dense nasopharyngeal mass obliterating both torus tubarius and fossae of Rosen Muller. The mass was bound laterally by the medial pterygoid muscles and measured 5.4 cm × 6.0 cm. There were no calcifications seen with in the mass. **B** A contrast enhanced axial CT at the same level and demonstrated the same features as on **A**. In addition, there was mild homogeneous enhancement of the mass. This was a diagnosis of nasopharyngeal carcinoma, made on both **A** and **B**

## Discussion

The purpose of this study was to determine the intra- and inter-observer agreement between paediatric head and neck malignancy findings of single CT procedures and double CT procedures for children aged 15 years and below at Uganda Cancer Institute. Majority of the images selected in the study belonged to children in the age range of 3 to 13 years with a median age of 9 years. We determined both inter-observer agreement as well as intra-observer agreement in the diagnosis of the tumours using the various CT protocols.

### Inter-observer agreement

#### Primary tumour location

When compared with previous literature, this study resonates with what has been previously reported by Just da Costa e Silva et al. [14]. The observed substantial agreement obtained on contrast enhanced CT only and unenhanced CT only procedures demonstrated that using either procedure provided the same value in regard to determining the primary tumour location. These findings resonate with reports in documented literature by Brennan et al. (2006) and Stambuk et al. (2005) [18, 19].

#### Tumour calcifications

This demonstrated that unenhanced CT only procedures did not offer a significant edge over contrast enhanced CT only procedures for detection of calcifications. This discordance was neither reflected in the diagnosis where a substantial agreement was obtained for contrast enhanced CT only procedures, unenhanced CT only procedures and double CT procedures. The findings from this study do resonate with the findings reported in previous literature [14] where they reported that contrast enhanced CT procedures had a sensitivity greater than 70% and specificity of 100% at detecting calcifications in paediatric abdominal malignancies.

#### Lymphadenopathy

Findings from this study demonstrated that there was better agreement on presence of lymphadenopathy with unenhanced CT only procedures as opposed to contrast enhanced CT only procedures which was different from reports in literature [20]. The explanation for these findings is unclear however may be explained by the intra-observer agreement findings for the different observers when contrast enhanced CT only procedures are compared to the findings on the double CT procedures.

#### Diagnosis

This demonstrated that using both unenhanced and contrast CT procedures did not provide an added advantage over using either contrast enhanced or unenhanced CT only procedures only. This is similar to the results presented by Just da Costa e Silva et al. [14] where diagnoses made on contrast enhanced CT only procedures were compared to histopathology results. For practice in our setting, this implies that contrast enhanced CT only procedures are sufficient in diagnosing PHNM.

### Intra-observer agreement

#### Primary tumour location

This demonstrated that either unenhanced or contrast enhanced CT only procedures are sufficient in determining the primary location of a tumour and using both does not offer an advantage over use of only one. The implication of this finding for clinical practice in our study is that patients with contraindications to contrast enhanced CT procedures and lack of access to MRI will still benefit from unenhanced CT procedures.

#### Tumour calcifications

Contrast enhanced CT only and unenhanced CT only procedures were comparable in terms of tumour calcification identification which is similar to what was reported by Just da Costa e Silva et al. [14]. The possible explanation for the differences among the observers could be due to lack of documentation of presence of calcifications while reporting the images which pertains to the presence of intrinsic differences among the observers [21, 22]. This may however also allude to the lack of importance of presence or absence of tumour calcifications when diagnosing PHNM. Indeed, this compares with what has been previously reported in literature by Lloyd et at. (2010) where presence of calcifications was only important in diagnosing retinoblastoma [23].

#### Lymphadenopathy

Observers A and C had an almost perfect intra-observer agreement when contrast enhanced CT only procedures were compared to double CT procedures as opposed to a substantial agreement when unenhanced CT only procedures were compared to double CT procedures, inferring that contrast enhanced CT only procedures are the preferred for demonstrating lymphadenopathy. Reports by Chong et al. [24], Restrepo et al. [25] and Som et al. [26] had similar conclusions. The fair intra-observer agreement by observer B may explain the moderate inter-observer agreement for lymphadenopathy demonstrated on contrast enhanced CT only procedures. Although observer A and C demonstrated a substantial agreement when unenhanced CT only procedures and double CT procedures were compared, metastatic spread to lymph nodes may occur in normal sized lymph nodes and contrast enhanced CT procedures provide an advantage as they delineate increased and heterogeneous enhancement of the lymph nodes unlike unenhanced CT [27].

#### Diagnosis

The apparent differences could be explained by intrinsic differences between the different observers and their years of experience [28, 29]. For observers B and C, there was no advantage offered by use of either contrast enhanced CT only procedures over use of unenhanced CT only procedures in making a diagnosis. Observer A, however, had a better agreement with use of contrast enhanced CT only procedures as opposed to unenhanced CT only procedures. This finding, which is similar to reports in literature, demonstrates that contrast enhanced CT only procedures have an advantage over unenhanced CT only studies in PHNM [6, 30, 31].

Findings from this study suggest key implications for practice particularly that in evaluation for paediatric head and neck malignancies, in a bid to reduce radiation exposure to patients, contrast enhanced only CT procedures are sufficient. The paediatric patients do not require unenhanced CT procedures for the sole purpose of identifying the tumour calcifications as radiologists are able to detect them on contrast enhanced CT only procedures. In consideration of these findings, radiology residents should be trained to report on paediatric head and neck malignancies with contrast enhanced CT only procedures.

Generally, the findings in this study do agree with previous literature, but the strength of the study lies in its being conducted in a resource-limited setting whose findings can be adaptable to many other resource-limited contexts. Another strength lies in using three observers of varying experiences, which offered a rich context to get opinion from different radiologists hence increasing the validity of the findings.

Despite the observed strengths of the study, there were some limitations. For example, all observers knew that the final diagnosis was a malignancy, which may have accounted for the substantial agreement on all protocols. Agreement for the diagnoses that the radiologists mentioned was determined only from the first diagnosis listed; however, in clinical practice; radiologists make a list of differential diagnoses, which are all considered. Intra- and inter-observer agreement on tumour size was not evaluated due to missing data from the observers. The study used a structured reporting template, as opposed to a checklist, which may have increased the chances of missing data. However, the study still provides useful findings that can inform clinical practice in similar settings. We do suggest more research particularly a level II phase III (multi-centre multi-observer) study, as suggested by Fryback and Thronbury [32], with the use of contrast enhanced paediatric head and neck CT only procedures and correlation with histopathology results is recommended to determine the generalizability of these results. 

## Conclusions

In our settings and examining a limited number of CT images, we demonstrated that contrast-enhanced CT scans provide sufficient information with no evidence of additional value of unenhanced images. Using contrast-enhanced images alone reduced the radiation exposure significantly.

The inter-observer agreement for diagnosis was substantial for contrast enhanced CT only, unenhanced CT only and double CT procedures when compared implying that contrast enhanced CT only procedures, unenhanced CT only procedures and double CT procedures may provide the same final diagnosis. Contrast enhanced CT only procedures are the preferred single CT procedure of choice as evidenced by a better intra-observer agreement for primary tumour location, lymphadenopathy and diagnosis. The patients being investigated for paediatric head and neck malignancies with iodine contrast allergies and/or renal failure can benefit from unenhanced CT procedures, which provide sufficient information on primary tumour location, presence of calcifications, presence of lymphadenopathy and a diagnosis.

## Data Availability

Local ethical guidelines do not permit submitting raw data, but the raw data is available from the corresponding author on individual request.

## Abbreviations

PHNM: Paediatric head and neck malignancies
CT: Computerized tomography
RISM: Radiation-induced secondary malignancies
